# Benzathine penicillin G stockouts and other barriers to documented syphilis treatment in pregnancy in Zambia

**DOI:** 10.1371/journal.pone.0304576

**Published:** 2024-06-03

**Authors:** Anna V. Jones, Albert Manasyan, Yumo Xue, Herbert Kapesa, Kate Mwendafilumba, Leukanji Nalwamba, Maureen Mzumara, Mwangelwa Mubiana-Mbewe, Jodie A. Dionne

**Affiliations:** 1 Heersink School of Medicine, University of Alabama at Birmingham, Birmingham, Alabama, United States of America; 2 Department of Pediatrics, University of Alabama at Birmingham, Children’s of Alabama Hospital, Birmingham, Alabama, United States of America; 3 Centre for Infectious Disease Research in Zambia, Lusaka, Zambia; 4 Center for Women’s Reproductive Health, University of Alabama at Birmingham, University of Alabama at Birmingham Hospital, Birmingham, Alabama, United States of America; 5 Department of Medicine, Division of Infectious Diseases, University of Alabama at Birmingham, University of Alabama at Birmingham Hospital, Birmingham, Alabama, United States of America; University of Cape Town, SOUTH AFRICA

## Abstract

**Objective:**

The prevalence of syphilis in Zambia remains high and is a critical public health concern. The Zambian Ministry of Health recommends universal screening and same-day treatment for syphilis in pregnancy, yet the syphilis screening rate is low, and treatment is poorly documented. The goal of this study was to document syphilis treatment rates and associated factors among pregnant women in care in Zambia.

**Methods:**

This retrospective cohort study included pregnant women diagnosed with syphilis according to rapid plasma reagin (RPR) screening during routine antenatal care (ANC) in Lusaka, Zambia in 2018–2019. The main outcome of interest was lack of documented BPG treatment during pregnancy. Additional information about pregnancy and neonatal outcomes, partner referral for therapy, and facility level stockout data were included. Patient characteristics were compared by treatment status using Pearson Chi-Square Test and logistic regression models were created to estimate the association between individual level-factors, facility type, and lack of BPG treatment. A Cochran-Mantel-Haenszel test was used to evaluate facility-level data with significance set at p<0.05.

**Results:**

Among 1,231 pregnant women who screened positive for syphilis at clinic, 643 (52%) lacked documented antibiotic treatment at the facility. BPG was the only antibiotic used to treat syphilis in the cohort and 8% of sex partners had evidence of referral for therapy. Preterm delivery rates were higher in women without documented BPG (43% vs 32%; p = 0.003). In adjusted models, only calendar year and hospital facility type were associated with lack of treatment. At the facility level, annual syphilis screening rates ranged from 37–65% and most (7/10) clinics reported at least one stockout of BPG.

**Conclusion:**

Treatment rates for syphilis in pregnancy in Zambia were low and BPG medication stockouts at the facility level were common. A consistent supply of BPG at all ANC facilities is needed to facilitate timely treatment and improve birth outcomes.

## Introduction

Congenital syphilis occurs when the bacterial spirochete *Treponema pallidum* crosses the placenta during pregnancy [1]. Up to 82% of women with untreated syphilis in pregnancy have adverse birth outcomes including stillbirth, preterm delivery, low birthweight, and neonatal death [2, 3]. Most infants with congenital syphilis lack clinical signs at birth but may develop later manifestations in the absence of treatment [4]. The clinical spectrum of congenital syphilis disease includes bony malformation, severe anemia, hepatosplenomegaly, jaundice, meningitis, visual changes, hearing loss, and skin rash with desquamation. Nearly all (97%) fetal and neonatal deaths from congenital syphilis can be prevented by early diagnosis and treatment with intramuscular benzathine penicillin (BPG) treatment in pregnancy, as recommended by national and global authorities for decades [5].

Barriers to the diagnosis and treatment of syphilis in pregnancy exist and persist in many countries. Treatment barriers vary by setting and are not well documented in sub-Saharan Africa (SSA) where syphilis is endemic. In recent reports, the prevalence of syphilis in pregnancy in Zambia was 4% [6]. In alignment with the World Health Organization (WHO), the Zambian Ministry of Health recommends universal screening and same-day treatment for syphilis in pregnancy [7]. Despite this, the syphilis ANC screening rate in Zambia was 48% in 2020 [6]. Routine sex partner notification and treatment is recommended but compliance is poorly documented [7, 8]. Barriers to treatment in pregnancy in low- and middle-income countries (LMIC) and the US include supply chain issues and BPG stockouts [9, 10]. To assess the syphilis treatment cascade in pregnancy in Zambia, we designed this study to assess individual-level and facility-level factors associated with a lack of documented BPG therapy. We believe that our study’s results may be generalizable to other SSA countries and LMIC settings with similar barriers to treatment.

## Materials and methods

### Study participants

This retrospective cohort study included pregnant women seen in ANC clinic in Lusaka, Zambia between January 1^st^, 2018 and December 31^st^, 2019 who screened positive for syphilis during a visit. Subsequent pregnancy and neonatal health outcomes were collected through June 30^th^, 2020. Individual-level variables in the dataset included age, HIV status, marital status, parity, timing of entry to ANC care, and estimated delivery date based on last menstrual period or ultrasound. We also collected information on the timing of syphilis screening, screening test type, receipt, and timing of syphilis treatment (BPG or alternative therapy) anytime during pregnancy, and sex partner referral for antibiotic therapy. ANC facility-level variables collected from monthly registers included syphilis and HIV screening rates, test kit supply and BPG supply with stockout duration captured in months (0–24).

### Study setting

In Zambia, about 55% of the population lives in poverty. Lusaka is the most populated Zambian province, with a population estimated at 2,888,575. Residents of Lusaka often live in squatter and unplanned settlements leading to inadequate access to housing, energy, clean drinking water, quality health services, and employment opportunities. As a country, Zambia has a high burden of disease that is characterized by high prevalence of HIV, sexually transmitted infections, tuberculosis, and malaria [11].

The study was conducted at ten public health facilities in urban Lusaka, Zambia including health centers and general hospitals where maternal and child health (MCH) services are provided. All facilities offered syphilis and HIV testing and treatment free of charge and most patients receive treatment and follow up care at the same ANC facility. General hospitals offer inpatient services and surgical services, including Caesarean deliveries, and have a wider catchment area compared to health centers. Laboratory services have comparable quality at all sites through MOH regulatory mechanisms. While it is possible that very few women accessing ANC services at general hospitals may be higher risk, this is not the majority. Many communities only have a general hospital and not a health center, thus they cater to all pregnant women irrespective of risk status. Higher risk pregnant women are mostly referred to major referral hospitals [12].

### Data collection

Data collection began in October 2021 and was completed in March 2022. Study data at the individual level for women diagnosed with syphilis was collected from routine medical records in paper format and the national electronic HIV health record system (SmartCare) for women living with HIV with deduplication. Birth outcomes were collected by certified research associates and community health workers through health record review and/or self-reported information obtained during household visits. This follow up information for pregnancy outcomes was available for a subset of women.

### Syphilis testing and treatment

According to routine clinical practice during the study period, syphilis screening was performed with a non-treponemal rapid plasma reagin (RPR) test (Omega Diagnostics, United Kingdom). Syphilis was defined according to a reactive RPR test and additional titer quantification. Confirmatory treponemal antibody testing was not performed. According to the local standard of care, all women who screened positive for syphilis were offered three weekly injections of BPG 2.4 million units to be administered in clinic. Receipt of BPG was defined in the medical record as any BPG treatment. Three doses of BPG 2.4 million units is standard recommended therapy but not all participants received 3 doses. Although 3 doses are recommended in the absence of staging, it is important to note that single dose BPG is considered appropriate therapy for primary, secondary, and early latent syphilis according to WHO and CDC [7, 13, 14].

### Statistical analysis

Demographic characteristics were reported as median and interquartile range (IQR) for continuous variables, and as number (proportion) for categorical variables, and compared according to documented syphilis treatment status in pregnancy using Wilcoxon rank-sum test, Pearson Chi-square test or Fisher’s Exact Test, as appropriate. Univariate and multivariable logistic regression models were used to estimate the association between individual level-factors, ANC facility type, and lack of documented BPG treatment as the main outcome of interest. The multivariable logistical regression model included variables with significance (p<0.05) in unadjusted models with results reported as odds ratios with 95% confidence intervals. A Cochran-Mantel-Haenszel test was used to evaluate facility-level data. All analyses were performed using SAS (version 9.4; SAS Institute; Cary, NC), with level of significance set at p<0.05 for all analyses. There was no imputation for missing data.

### Ethics and consent

This investigation was approved by the University of Zambia Biomedical Research Ethics Committee (Ref #: 1801–2021) and the University of Alabama at Birmingham Institutional Review Board (IRB-300007767). Permission to review individual patient data was approved from the relevant local and international IRBs through protocol review procedures under the National Health Research Authority. All staff who had contact with participants received training on the protection of human research participants prior to conducting study activities. The confidentiality of all study records was safeguarded with unique coded participant ID numbers.

## Results

Our cohort included 1,231 unique pregnant women diagnosed with syphilis in ANC clinic. Based on facility level data, 4.8% of women screened positive (1,374/28,480). Median age of the cohort was 27 years old, 43% were living with HIV, 73% had established ANC care between 15–28 weeks gestational age, and 28% had parity ≥3. In terms of timing, nearly all (99.7%) were screened for syphilis at their entry to ANC, as recommended. A higher proportion attended clinic at a general hospital (58%) vs health center (42%) facility. All women with documented treatment received first line BPG therapy with no alternative antibiotics prescribed. Sex partner referral for therapy was infrequent (8%) and more common in those with documented BPG (14% vs 2%; p<0.001). Among 861 women with birth outcome information (861/1231; 70%), preterm delivery rates were more common in women without BPG (43% vs 32% with BPG; p = 0.002) and the proportion with low birthweight was similar (14% vs 10%; p = 0.07). Only 47% of women had documented BPG treatment. (Table 1)

**Table 1:** Characteristics of pregnant women with syphilis by treatment status.

	BPG Treatment	No Documented BPG Treatment	Total	
			n = 1,231	
	Documented	n = 642	median (IQR)	
	n = 589	median (IQR)	or n (%)	p-value
Variable	median (IQR)	or n (%)		
	or n (%)			
**Sociodemographic Variables**				
Age (years) (n = 1218)	27.0 (23.0,32.0)	27.0 (23.0,32.0)	27.0 (23.0,32.0)	0.75
Marital Status (n = 1156)				0.94
Married/Living with Partner	506 (88.3)	514 (88.2)	1020 (88.2)	
Single	67 (11.7)	69 (11.8)	136 (11.8)	
Age Categories (yrs) (n = 1218)				0.77
16–19	29 (4.9)	37 (5.9)	66 (5.4)	
20–24	161 (27.4)	156 (24.8)	317 (26.0)	
25–29	182 (30.9)	204 (32.4)	386 (31.7)	
30–34	118 (20.1)	121 (19.2)	239 (19.6)	
35+	98 (16.7)	112 (17.7)	210 (17.3)	
Positive HIV Status (n = 1204)	238 (41.1)	274 (43.8)	512 (42.5)	0.34
**Obstetric and Prenatal Care History**				
Parity (n = 1201)				0.59
0	130 (22.3)	118 (19.1)	248 (20.6)	
1–2	292 (50.0)	326 (52.9)	618 (51.5)	
3–4	132 (22.6)	142 (23.0)	274 (22.8)	
5+	30 (5.1)	31 (5.0)	91 (5.1)	
Gestational Age at Entry to ANC (n = 1088)				0.57
4–14 weeks	99 (18.5)	110 (19.9)	209 (19.2)	
15–28 weeks	395 (74.0)	395 (71.3)	790 (72.6)	
≥ 28 weeks	40 (7.5)	49 (8.8)	89 (8.2)	
ANC Calendar Year (n = 1228)				<0.001
2018	443 (75.2)	331 (51.8)	774 (63.0)	
2019	146 (24.8)	308 (48.2)	454 (37.0)	
Facility Type (n = 1231)				<0.001
Health Center	282 (47.9)	240 (37.4)	522 (42.4)	
General Hospital	307 (52.1)	402 (62.6)	709 (57.6)	
Number BPG Doses Received (n = 1231)				<0.001
0	0	642 (100)	642 (52.6)	
1	147 (25.5)	0	147 (12.1)	
2	23 (4.0)	0	23 (1.9)	
3	407 (70.5)	0	407 (33.4)	
Syphilis Test at ANC Entry (n = 1228)	588 (99.8)	636 (99.5)	1224 (99.7)	0.63*
Sex Partner Referral for Therapy (n = 1231)	85 (14.4)	15 (2.3)	100(8.1)	<0.001
**Adverse Birth Outcomes**				
Low Birthweight (<2500 gm)				0.07
(n = 837)	40 (9.9)	61 (14.1)	101 (12.1)	
Preterm delivery (<37 weeks) (n = 861)	121 (32.2)	171 (42.8)	292 (37.6)	0.002

Models created to assess factors associated with lack of documented BPG in pregnancy are shown in Table 2. Two factors were associated: calendar year at entry to ANC care (adjusted OR (aOR) 2.7 for 2019 vs 2018; 95% CI 2.1–3.4; p<0.001) and hospital facility type (aOR 1.36 vs health centers; 95% CI 1.08–1.72; p = 0.01).

**Table 2:** Factors associated with lack of documented BPG in pregnancy (n = 1231).

Variable	Unadjusted Odds Ratio (95% CI)	p-value	Adjusted Odds Ratio (95% CI)	p-value
Age Categories		0.77		
16–19	1.12 (0.64,1.95)			
20–24	0.85 (0.60,1.20)			
25–29	0.98 (0.70,1.37)			
30–34	0.90 (0.62,1.30)			
35+	REF			
Parity		0.18		
0	REF			
1+	1.21 (0.92,1.60)			
Marital Status		0.94		
Married/Living with Partner	REF			
Single	1.01 (0.71,1.45)			
HIV		0.34		
Negative	REF			
Positive	1.12 (0.89,1.41)			
Timing of Entry to Care		0.57		
4–13 weeks	REF			
14–27 weeks	0.90 (0.66,1.22)			
28+ weeks	1.10 (0.67,1.81)			
Year at Entry to Care		<0.001		<0.001
2018	REF		REF	
2019	2.82 (2.21,3.60)		2.71 (2.11,3.47)	
ANC Facility Type				0.011
Health Center	REF	<0.001	REF	
General Hospital	1.54 (1.23,1.93)		1.35 (1.07,1.71)	

Table 3 compares annual facility level syphilis screening and HIV screening rates. The syphilis screening rate during the study period was 43% and the HIV screening rate was 88%. Although the syphilis screening rate was higher at health centers compared to hospitals in 2018 (65% vs 40%; p<0.001), screening rates by facility type were similarly low in 2019 (39% vs 37%). In contrast, the HIV screening rate held steady at 88% overall with slightly higher screening rates at hospitals compared to health centers in 2019 (88% vs 84%; p<0.001).

**Table 3:** Facility level syphilis and HIV screening rates.

Characteristic	Hospital	Health Center	Total	p-value
**Syphilis Screening Rate**				<0.001
2018	8066/19,958 (40.4)	7495/11,577 (64.7)	15,561/31,535 (49.3)	
2019	8391/22,754 (36.9)	4528/11,487 (39.4)	12,919/34,241 (37.7)	
**HIV Screening Rate**				<0.001
2018	17,618/19,958 (88.3)	10,274/11,577 (88.7)	27,892/31,535 (88.4)	
2019	20,101/22,754 (88.3)	9,708/11,487 (84.5)	29,809/34,241 (87.1)	

The frequency and duration of syphilis/HIV test kit and BPG treatment stockouts by facility type is shown in Table 4. All facilities reported syphilis test kit stockouts lasting at least one month (100%; 10/10). In contrast, HIV test kit stockouts were infrequent (10%; 1/10). Most facilities (70%) also reported BPG treatment stockouts. For BPG stockout duration, 60% (6/10) reported stockouts lasting 1–6 months and at one site, there was a prolonged BPG medication stockout lasting 13–24 months.

**Table 4:** Stockouts by facility type.

Characteristic	Hospital	Health Center	Total
	(n = 5)	(n = 5)	(n = 10)
	n (%)	n (%)	n (%)
**Any Syphilis Test Stockout**	5/5 (100)	5/5 (100)	10/10
**Test Stockout Duration (months)**			
0	0/5 (0)	0/5 (0)	0/10 (0)
1–6	0/5 (0)	2/5 (40)	2/10 (20)
7–12	3/5 (60)	3/5 (60)	6/10 (60)
13–24	2/5 (40)	0/5 (0)	2/10 (20)
**Any HIV Test Stockout**	1/5 (20)	0/5 (0)	1/10 (10)
**Penicillin (BPG) Stockout**	3/5 (60)	4/5 (80)	7/10 (70)
**BPG Stockout Duration (months)**			
0	2/5 (40)	1/5 (20)	3/10 (30)
1–6	2/5 (40)	4/5 (80)	6/10 (60)
7–12	0/5 (0)	0/5 (0)	0/10 (0)
13–24	1/5 (20)	0/5 (0)	1/10 (10)

## Discussion

Syphilis in pregnancy is a curable sexually transmitted infection when it is treated in a timely fashion with BPG, but treatment barriers exist. In this cohort study conducted in 10 clinics, only one in two pregnant women in Zambia diagnosed with syphilis had subsequent documentation of BPG treatment during pregnancy. This finding is consistent with published low treatment rates in other countries [15]. Published syphilis treatment rates in pregnancy in various settings have ranged from 24% in Nigeria and 50% in India in 2012, to 86% in China during 2015–2018 [15, 16]. In this study, we found no individual level predictors to be associated with lack of documented treatment, yet facility level factors and calendar year of care were associated. BPG stockouts occurred at most ANC sites which likely drove the association we identified between a lack of BPG treatment according to calendar year and facility type. BPG supply is a critical component of the syphilis treatment cascade in pregnancy and a modifiable barrier to meeting recommended global and national ANC care standards.

Lack of access to antibiotics due to supply chain disruptions is a complex and challenging global issue with increasing awareness since the COVID-19 pandemic [10]. Unfortunately, the global BPG supply chain is unreliable since few pharmaceutical companies manufacturer the necessary ingredients. As syphilis rates continue to rise, the current supply has been unable to meet clinical demand in high-income countries as well as LMIC settings. Further, appropriate use of antibiotics for all infections in Lusaka is low, further contributing to supply and demand issues [17]. As one strategy to improve the supply chain, the Zambian MOH has proposed a shift to local BPG production to ensure a more consistent supply. Additionally, antibiotic stewardship programs need to be strengthened to help combat inappropriate antibiotic usage affecting national supply.

It is well-established that untreated syphilis is associated with adverse pregnancy outcomes. In our study, we found evidence of higher preterm delivery rates among women without documented BPG [18]. Preterm birth is a leading cause of infant morbidity and mortality globally [19]. In addition to modifiable components of the syphilis treatment cascade, it is also important to consider the risk of syphilis reinfection during pregnancy after appropriate treatment. Unfortunately, among pregnant women diagnosed with syphilis in this study, few sex partners had evidence of referral for antibiotic therapy. This suggests that study participants may have remained at risk of syphilis reinfection. Novel efforts to increase sex partner referral and ensure linkage to timely syphilis treatment are urgently needed.

Despite longstanding universal screening recommendations, the syphilis screening rate in ANC care in our study was only 43%. Other studies in Zambia have reported syphilis screening rates ranging between 11%-28% [20, 21] while screening rates in Uganda and Nigeria averaged 40% [18]. In contrast, the ANC syphilis screening rate in South Africa is 75% which may reflect better access to care or testing supplies [18]. In terms of the timing of ANC syphilis screening in Zambia, nearly all women included in this study were screened in a timely manner at their first ANC visit. This important and beneficial finding reflects ongoing efforts by the Zambian MOH to increase syphilis screening rates at entry to care.

We documented a stark difference in the ANC screening rate for syphilis (43%) compared to HIV (88%). Study sites reported infrequent HIV test stockouts, while every study site reported syphilis test kit stockouts during the study period. As part of a national HIV strategy, the Zambian Ministry of Health prioritized HIV screening in pregnancy and allocated health funding from PEPFAR and other sources to increase the availability of HIV testing kits. Lastly, 43% of our cohort were coinfected with HIV and syphilis, compared to other studies in SSA where the HIV and syphilis coinfection rate in pregnant women ranged from 1–10% [22–24]. Our high coinfection rate reflects the increasing seropositivity in Zambia as reported in the MoH report and added vulnerability for women and infants since HIV and syphilis coinfection has been shown to exacerbate adverse birth outcomes [6]. In the past year, the Zambian MOH has initiated a nationwide scale-up of affordable HIV/Syphilis Dual testing with >95% sensitivity and specificity. This new program designed to align ANC syphilis and HIV testing rates should be assessed based on impact on treatment rates and outcomes.

The 4.8% syphilis seropositivity rate in this study is consistent with published rates in Zambia ranging from 1%-4% [20, 25, 26]. Syphilis positivity rates in other SSA countries range from 0.1% in Benin and 10% in Liberia [18, 24].

Study limitations include the retrospective design with outcomes limited to documented treatment and stockouts according to available paper-based government ANC records. Bias due to missing information is possible and true treatment rates may have been higher than documented BPG rates in the record. However, local providers in Zambia informed us that syphilis treatment outside of the ANC facility is uncommon. Since the study included only urban ANC clinics, findings may not generalize to rural ANC clinics although medication stockouts tend to have a nationwide impact. Also, syphilis in pregnancy could have been under- or over-diagnosed since testing was limited to RPR testing without confirmatory treponemal antibody testing. The association of preterm delivery with lack of documented BPG supports our categorization of treatment status. Those who were less likely to have documented BPG treatment might have also been less likely to have partner referral therapy or birth outcomes recorded. Study strengths include the large sample size across a wide area in a high-prevalence setting with relevant variables collected at the individual-level, facility-level, and community-level. It is a global priority to ensure a consistent supply of BPG not only in Zambia but in other countries with a high burden of syphilis. All facilities that provide ANC care should have an adequate and consistent supply of medication on hand.

## Conclusion

Syphilis in pregnancy in Zambia remains to be a critical public health concern in Zambia. Treatment rates for syphilis diagnosed in pregnancy in Zambia were inadequate at 47% and adverse birth outcomes such as preterm birth were associated with lack of documented treatment. Stockouts of syphilis test kits and benzathine penicillin G therapy occurred frequently at the facility level, further contributing to inadequate treatment. Novel approaches to ensure consistent access to syphilis testing and treatment in ANC clinic are urgently needed.

## Supporting information

S1 Dataset Unidentified raw data set used for this manuscript. (CSV)

## References

[1] Kimball A, Torrone E, Miele K, Bachmann L, Thorpe P, Weinstock H, et al. Missed Opportunities for Prevention of Congenital Syphilis—United States, 2018. MMWR Morb Mortal Wkly Rep. 2020;69(22):661–5. doi: 10.15585/mmwr.mm6922a1 32497029 PMC7272112

[2] Gomez GB, Kamb ML, Newman LM, Mark J, Broutet N, Hawkes SJ. Untreated maternal syphilis and adverse outcomes of pregnancy: a systematic review and meta-analysis. Bull World Health Organ. 2013;91(3):217–26. doi: 10.2471/BLT.12.107623 23476094 PMC3590617

[3] Wijesooriya NS, Rochat RW, Kamb ML, Turlapati P, Temmerman M, Broutet N, et al. Global burden of maternal and congenital syphilis in 2008 and 2012: a health systems modelling study. Lancet Glob Health. 2016;4(8):e525–33. doi: 10.1016/S2214-109X(16)30135-8 27443780 PMC6759483

[4] International Clearinghouse for Birth Defects Surveillance and Research (ICBDSR), National Center on Birth Defects and Developmental Disabilities from the United States Centers for Disease Control and Prevention (CDC), World Health Organization (WHO). Quick Reference Handbook of Selected Congenital Anomalies and Infections. 2020.

[5] Blencowe H, Cousens S, Kamb M, Berman S, Lawn JE. Lives Saved Tool supplement detection and treatment of syphilis in pregnancy to reduce syphilis related stillbirths and neonatal mortality. BMC Public Health. 2011;11 Suppl 3:S9. doi: 10.1186/1471-2458-11-S3-S9 21501460 PMC3231915

[6] Health RoZMo. Annual Health Statistical Report 2017–2020. Lusaka, Zambia; 2021.

[7] WHO Guidelines Approved by the Guidelines Review Committee. WHO Guideline on Syphilis Screening and Treatment for Pregnant Women. Geneva: World Health Organization. Copyright © World Health Organization 2017.; 2017. 29757595

[8] Health RoZMo. ANC guidelines for a positive pregnancy experience.

[9] Dassah ET, Adu-Sarkodie Y, Mayaud P. Rollout of rapid point of care tests for antenatal syphilis screening in Ghana: healthcare provider perspectives and experiences. BMC health services research. 2018;18(1):130. doi: 10.1186/s12913-018-2935-y 29458363 PMC5819248

[10] Nurse-Findlay S, Taylor MM, Savage M, Mello MB, Saliyou S, Lavayen M, et al. Shortages of benzathine penicillin for prevention of mother-to-child transmission of syphilis: An evaluation from multi-country surveys and stakeholder interviews. PLoS Med. 2017;14(12):e1002473. doi: 10.1371/journal.pmed.1002473 29281619 PMC5744908

[11] Planning MoND. Seventh National Development Plan 2017–2021. Lusaka, Zambia; 2017.

[12] Services ZNMaN. Referral Guidelines. Republic of Zambia Ministry of Health; 2018.

[13] Elimination of Mother-to-Child Transmission of HIV and Syphilis National Operational Plan 2019–2021. The Republic of Zambia Ministry of Health; 2019.

[14] Workowski KA, Bachmann LH, Chan PA, Johnston CM, Muzny CA, Park I, et al. Sexually Transmitted Infections Treatment Guidelines, 2021. MMWR Recomm Rep. 2021;70(4):1–187. doi: 10.15585/mmwr.rr7004a1 34292926 PMC8344968

[15] Chen XS, Khaparde S, Prasad TL, Srinivas V, Anyaike C, Ijaodola G, et al. Estimating disease burden of maternal syphilis and associated adverse pregnancy outcomes in India, Nigeria, and Zambia in 2012. Int J Gynaecol Obstet. 2015;130 Suppl 1:S4–9. 10.1016/j.ijgo.2015.04.014 25975870

[16] Li H, Tan J, Luo Z, Zheng J, Huang G, Xiao J, et al. Standardized treatment and determinants on 9,059 syphilis-infected pregnant women during 2015–2018 in Hunan, China. Sci Rep. 2020;10(1):12026. doi: 10.1038/s41598-020-69070-3 32694571 PMC7374608

[17] Ngoma MT, Sitali D, Mudenda S, Mukuma M, Bumbangi FN, Bunuma E, et al. Community antibiotic consumption and associated factors in Lusaka district of Zambia: findings and implications for antimicrobial resistance and stewardship. JAC Antimicrob Resist. 2024;6(2):dlae034. doi: 10.1093/jacamr/dlae034 38449513 PMC10914457

[18] Kuznik A, Habib AG, Manabe YC, Lamorde M. Estimating the Public Health Burden Associated With Adverse Pregnancy Outcomes Resulting From Syphilis Infection Across 43 Countries in Sub-Saharan Africa. Sex Transm Dis. 2015;42(7):369–75. doi: 10.1097/OLQ.0000000000000291 26222749 PMC4520246

[19] Griffin JB, Jobe AH, Rouse D, McClure EM, Goldenberg RL, Kamath-Rayne BD. Evaluating WHO-Recommended Interventions for Preterm Birth: A Mathematical Model of the Potential Reduction of Preterm Mortality in Sub-Saharan Africa. Glob Health Sci Pract. 2019;7(2):215–27. doi: 10.9745/GHSP-D-18-00402 31249020 PMC6641817

[20] Berrueta M, Cafferata ML, Mwenechanya M, Nkamba Mukadi D, Althabe F, Bergel E, et al. Syphilis screening and treatment in pregnant women in Kinshasa, Democratic Republic of the Congo and in Lusaka, Zambia: a cross-sectional study. Gates open research. 2017;1:13. doi: 10.12688/gatesopenres.12768.1 29355227 PMC5764227

[21] Bonawitz RE, Duncan J, Hammond E, Hamomba L, Nambule J, Sambambi K, et al. Assessment of the impact of rapid syphilis tests on syphilis screening and treatment of pregnant women in Zambia. Int J Gynaecol Obstet. 2015;130 Suppl 1(Suppl 1):S58–62. doi: 10.1016/j.ijgo.2015.04.015 25968492 PMC8991823

[22] Acosta LMW, Gonçalves TR, Barcellos NT. [HIV and syphilis coinfection in pregnancy and vertical HIV transmission: a study based on epidemiological surveillance data]. Rev Panam Salud Publica. 2016;40(6):435–42. 28718492

[23] Yeganeh N, Watts HD, Camarca M, Soares G, Joao E, Pilotto JH, et al. Syphilis in HIV-infected mothers and infants: results from the NICHD/HPTN 040 study. Pediatr Infect Dis J. 2015;34(3):e52–7. doi: 10.1097/INF.0000000000000578 25742089 PMC4352722

[24] Manyahi J, Jullu BS, Abuya MI, Juma J, Ndayongeje J, Kilama B, et al. Prevalence of HIV and syphilis infections among pregnant women attending antenatal clinics in Tanzania, 2011. BMC Public Health. 2015;15:501. doi: 10.1186/s12889-015-1848-5 25994129 PMC4492104

[25] Larson BA, Lembela-Bwalya D, Bonawitz R, Hammond EE, Thea DM, Herlihy J. Finding a needle in the haystack: the costs and cost-effectiveness of syphilis diagnosis and treatment during pregnancy to prevent congenital syphilis in Kalomo District of Zambia. PLoS One. 2014;9(12):e113868. doi: 10.1371/journal.pone.0113868 25478877 PMC4257564

[26] Althabe F, Chomba E, Tshefu AK, Banda E, Belizán M, Bergel E, et al. A multifaceted intervention to improve syphilis screening and treatment in pregnant women in Kinshasa, Democratic Republic of the Congo and in Lusaka, Zambia: a cluster randomised controlled trial. Lancet Glob Health. 2019;7(5):e655–e63. doi: 10.1016/S2214-109X(19)30075-0 30910531 PMC6465956

